# MeCP2 Modulates Depression‐Like Behaviors Comorbid to Chronic Pain by Regulating Adult Hippocampal Neurogenesis

**DOI:** 10.1111/cns.70311

**Published:** 2025-04-07

**Authors:** Yanting Sun, Ying Zhang, Yexiang Chen, Huisheng Peng, Tiantian Cheng, Xiujian Sun, Jing‐Gen Liu, Chi Xu

**Affiliations:** ^1^ Key Laboratory of Acupuncture and Neurology of Zhejiang Province, Department of Neurobiology and Acupuncture Research The Third Affiliated Hospital of Zhejiang Chinese Medical University Zhejiang Hangzhou China; ^2^ School of Pharmaceutical Sciences Zhejiang Chinese Medical University Zhejiang Hangzhou China

**Keywords:** adult neurogenesis, chronic pain, depression, MeCP2, microRNA

## Abstract

**Aims:**

Although previous studies have revealed the association between chronic pain‐induced depression and defective adult hippocampal neurogenesis (AHN), the underlying molecular mechanism remains elusive. This study aims to examine the association between AHN and depression‐like behaviors, and to reveal the underlying mechanisms.

**Methods:**

The chronic neuropathic pain model was established using mice with the spared nerve injury (SNI) surgery. The depression‐like behaviors were evaluated by using the sucrose preference test (SPT), the tail suspension test (TST), the forced swimming test (FST), and the open field test (OFT). The expression of Methyl‐CpG‐binding protein 2 (MeCP2) was modulated by injecting the adeno‐associated virus (AAV) with the DIO system into the ventral DG of the *Nes*‐CreER^T2^ mice. The miRNAs in hippocampal neural stem cells (NSCs) of mice with chronic pain were analyzed via miRNA sequencing.

**Results:**

We found that MeCP2, an epigenetic factor that plays a key role in the development of neurons, was significantly down‐regulated in NSCs in the dentate gyrus (DG) of the hippocampus in adult mice with chronic pain and comorbid depression, suggesting a role of MeCP2 in the regulation of depression‐like behavior induced by chronic neuropathic pain. MeCP2 expression levels in hippocampal NSCs were closely related to AHN and chronic pain comorbid depression, and miR‐199b‐3p specifically targeted and inhibited MeCP2 expression by directly interacting with its 3’‐UTR sequence. Furthermore, we demonstrated that the increased level of miR‐199b‐3p in NSCs after the occurrence of chronic pain was responsible for AHN inhibition and comorbid depression.

**Conclusion:**

Chronic neuropathic pain may result in an increased level of miR‐199b‐3p in hippocampal NSCs, which in turn targeted the *Mecp2* gene and inhibited its transcription. Inhibited MeCP2 expression in NSCs contributes to AHN inhibition and depression‐like behaviors.

## Graphical abstract

Downregulation of MeCP2 targeted by miR‐199‐3p in the hippocampal neural stem cells of mice with chronic neuropathic pain induces inhibition of adult neurogenesis, thus leading to depression‐like behaviors.

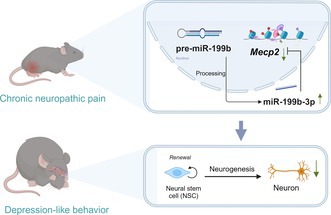

## 1 Introduction

Chronic pain is a multidimensional sensational and emotional experience involving sensory discrimination characterized by spontaneous pain (persistent, episodic) and evoked types of pain (nociceptive hypersensitivity, tenderness), along with affective motivation and cognitive appraisal [1]. More than half of the patients with chronic pain suffer from comorbid negative emotions, such as depression and anxiety, which may, in turn, exacerbate pain sensitivity, thus forming a vicious circle [2, 3, 4]. Depression is a major concern among the negative emotions accompanying chronic pain, and the current management for chronic pain comorbid with depression mainly relies on medications including selective 5‐hydroxytryptamine reuptake inhibitors, tricyclic antidepressants, tetracyclic antidepressants, and monoamine oxidase inhibitors [5]. However, the efficacy of these antidepressants in ameliorating pain‐related depression remains limited. During the past decade, adult hippocampal neurogenesis (AHN) has been recognized as a ubiquitous process in the mammalian brain, with close association with both sensational and emotional processes [6]. Thus, new mechanisms underlying adult neurogenesis may be crucial for exploring molecular targets for the treatment of chronic pain comorbid with depression.

AHN is a process through which the neural stem cells (NSCs) in the dentate gyrus (DG) undergo neuronal differentiation and generate neuroblasts, which, in turn, develop into mature granule cells and incorporate into the hippocampal neural circuit [6, 7]. This process has been proven to play a key role in the regulation of pain signals in the brain [8], along with psychiatric and cognitive activities, such as learning, memory, and emotions [9]. A marked inhibition of AHN was discovered in company with depression‐like behavior, which can be effectively alleviated by enhancing AHN [10]. On the other hand, altered AHN was also detected in mice with chronic neuropathic pain, accompanied by undermined synaptic plasticity [8]. The previous study has confirmed the effectiveness of enhanced AHN in ameliorating chronic pain comorbid depression, thus inspiring a new strategy in the treatment of pain‐induced depression [6], which relies on a comprehensive understanding of the underlying mechanisms.

MicroRNAs (miRNAs) are small noncoding RNAs with 16–28 nucleotides in length that are first transcribed into primary (pri‐) miRNAs, which are processed by the Drosha enzyme to form precursor (pre‐) miRNAs in the nucleus and transported to the cytoplasm, where they are cleaved by Dicer during maturation [11]. MiRNAs inhibit the expression of target genes by binding complementarily to the 3′ UTR of the target mRNA, thus inhibiting the translation process by leading to mRNA degradation [12]. MicroRNAs play important roles in the central nervous system by modulating a series of physiological processes including AHN [13, 14, 15], thus participating in the development and plasticity of the nervous system, as well as in the onset and development of neurodegenerative diseases [16].

Methylated CpG binding protein 2 (MeCP2) is a nuclear protein considered as a key transcriptional modulator of genes expressed in mammalian neurons, which binds to the methylated sites of its target genes and inhibits their transcription, on both transcriptional and posttranscriptional levels [17]. Due to its ubiquitous and high‐level expression in neurons of the central nervous system [18], MeCP2 is able to affect the expression of genes required for long‐term plasticity associated with neuronal activity by regulating the structure of chromatin, thus affecting both cognitive and emotional functions [19]. Mutations in MeCP2 are found in a variety of neural disorders, such as Rett's syndrome [20], depression, addiction, and schizophrenia [21, 22]. Moreover, MeCP2 may directly control miRNA processing, either by its phosphorylation [23], or by binding to the RNA‐binding domain of the DiGeorge syndrome critical region gene (DGCR) [24]. Such MeCP2/miRNA interactions have been proven to regulate cell fate determination in NSC differentiation [25] and neurogenesis [26]. Thus, in the present study, we focused on the effect of miRNA/MeCP2 on AHN in mice with depression comorbid to chronic neuropathic pain and elucidated the mechanism by which AHN and depression‐like behavior were controlled.

## 2 Materials and Methods

### 2.1 Animals

Six‐week‐old male C57BL/6J mice were from the Shanghai Laboratory Animal Center, Chinese Academy of Sciences, and the *Nes*‐CreER^T2^ transgenic mice (full name: C57BL/6‐Tg (*Nes*‐Cre/ERT2) KEisc/J; RRID: IMSR_JAX: 016261) were from the Jackson Laboratory (US). The induction of Cre recombinase was performed according to the originating study [27], with intraperitoneal injections of tamoxifen (Merck, Germany) at 180 mg/kg/day for five consecutive days. All animals were housed under standard environmental conditions (12‐h light–dark cycle and 24°C ± 2°C) with access to food and water provided ad libitum, in the Laboratory Animal Center of Zhejiang Chinese Medical University accredited by the Association for Assessment and Accreditation of Laboratory Animal Care (AAALAC). All animals were euthanized immediately after the accomplishment of experiments by euthanizing, and all experimental procedures were approved by the Animal Ethics Committee of Zhejiang Chinese Medical University (permission number: IACUC‐20220328‐09) and conducted according to the National Institutes of Health Guide for the Care and Use of Laboratory Animals (NIH Publications No. 80‐23).

### 2.2 SNI Model

The SNI model was prepared according to the previous study [6], with slight modifications. Mice were anesthetized with 2% isoflurane inhalation. The left hind limb sciatic nerve was exposed, and the sural and common peroneal branches were tightly ligated with nonabsorbent 6‐0 sutures. For the sham group, the sciatic nerve was exposed without further manipulation.

### 2.3 Mechanical Pain Thresholds

Mechanical pain thresholds were tested using the von Frey filament test as previously described [6]. The filament probe was applied perpendicularly to the central plantar surface of the hind paw, and the responses of von Frey hairs (Stoelting, US) with different sizes were measured using the up‐and‐down method. Paw withdrawal, flinching, and licking of the claws were considered positive responses. The 50% paw withdrawal threshold (PWT) was calculated using the following formula: 50% PWT (g) = 10Xf + *k*δ /10,000, where Xf is the handle mark value of the last von Frey hair used, *k* is the tabular value for the pattern of positive/negative responses, and *δ* is the average interval (in log units) between von Frey hairs.

### 2.4 Microdissection of DG

Mouse DG was collected as previously described [6]. The brains of mice that experienced the whole process of behavioral tests were collected in 20‐mL cold Hank's balanced salt solution (HBSS, Thermo Fisher Scientific, US), cut into 400‐μm coronal sections using a double blade, and collected in a 6‐cm Petri dish. The whole procedure was performed on ice. The ventral DG tissue (−2.5~−4.0 mm AP) was collected using a cryostat slicer (Thermo Fisher Scientific).

### 2.5 Quantitative Reverse Transcription PCR (qRT‐PCR)

All mice used for qRT‐PCR were from those that experienced the whole process of behavioral tests. After the euthanization of the mice, the DG samples were collected, and the MiniBEST Universal RNA Extraction Kit (Takara, Japan) was used to extract total RNA samples. The cDNA samples were prepared using PrimeScript RT Reagent Kit (Takara) and the LightCycler 480 System (Roche, Switzerland) was used for quantitative PCR, with the TB Green Fast qPCR Mix (Takara). *Actb* (β‐actin) was used as a reference gene. The primer sets are listed below:

*Actb* forward: 5′‐TCCTCCCTGGAGAAGAGCTA‐3′

*Actb* reverse: 5′‐CCAGACAGCACTGTGTTGGC‐3′

*Nestin* forward: 5′‐GCTACATACAGGACTCTGCTGG‐3′

*Nestin* reverse: 5′‐GGTGCTGGTCCTCTGGTATC‐3′’

*Mcep2* forward: 5′‐CCAGGCTTTCTACCCCGTTT‐3′

*Mcep2* reverse: 5′‐CTGCCCAGGTCATGGTGATC‐3′

*Dcx* forward: 5′‐CCTTGGATGAGAATGAATGC‐3′

*Dcx* reverse: 5′‐TTTGCGTCTTGGTCGTTA‐3′

*Olig2* forward: 5′‐CGTTAACACGAGGGGCAAAC‐3′

*Olig2* reverse: 5′‐CGGAAGAGGTGGAAGGTTAGG‐3′

*Gfap* forward: 5′‐GTCTAAGTTTGCAGACCTCACAGA‐3′

*Gfap* reverse: 5′‐GACTCCAGATCGCAGGTCAAG‐3′

### 2.6 Western Blot Assay

All mice used for western blot were from those that experienced the whole process of behavioral tests. After the euthanization of the mice, the supernatants were collected from homogenized mouse DG samples. The protein concentrations were quantified with the bicinchoninic acid (BCA) protein quantification kit (Abcam, UK) according to the manufacturer's instructions. The protein concentration of all samples was adjusted to the same level, with the total protein of each loaded sample limited to 10–20 μg to alleviate the effect of membrane saturation that may cause inconsistency of internal controls with the actual values [28, 29]. The samples were mixed with a loading buffer that contained 2% SDS, 100 mM dithiothreitol, 10% glycerol, and 0.25% bromophenol blue, and the mixture was then separated on a 10% SDS–PAGE gel and electrophoretically transferred onto polyvinylidene difluoride (PVDF) membranes (Merck, Germany). Chemiluminescence was detected with ECF reagents (GE Healthcare, UK) and visualized using an ImageQuant LAS 4000 (GE Healthcare), and protein bands were quantified using Quantity One 1D analysis software (Bio‐rad, US). Antibodies used are listed in Table S1.

### 2.7 Stereotaxic Microinjection of the Adeno‐Associated Virus (AAV)

The mice were anesthetized with 1% pentobarbital sodium, and holes of 0.5 mm diameter were drilled on both sides of the skull. A total volume of 500 nL virus (250 nL for each side) was infused into the ventral DG (−3.3 mm AP, ±2.3 mm ML, and −2.1 mm DV from the bregma) at a constant rate of 100 nL/min. The mice were left in the same position for 8 min after the injection to allow virus infusion.

The following AAV constructs (BrainVTA, China) were used in this study:

rAAV‐EF1α‐DIO‐*Mecp2*‐EGFP‐3× FLAG was used for MeCP2 overexpression in NSCs, rAAV‐EF1α‐DIO‐EGFP‐Sh*Mecp2*‐WPRE‐Hgh polyA was used for MeCP2 knockdown in NSCs, and rAAV‐hSyn‐DIO‐EGFP‐WPRE‐Sh‐miR‐199b‐3p‐hGH polyA was used for miR‐199b‐3p knockdown in NSCs.

### 2.8 Bromodeoxyuridine (BrdU) Labelling, Immunohistochemistry, and In Vivo Cell Quantification

All mice used for immunohistochemistry were from those that experienced the whole process of behavioral tests. The mice were intraperitoneally injected with BrdU (Merck, Germany) once daily at a dose of 150 mg/kg/day for three consecutive days. After euthanization, mouse brains were collected following perfusion with 0.9% saline and then 4% paraformaldehyde. Coronal sections measuring 30 μm thick were produced by using a Thermo Fisher Scientific cryostat on the ventral hippocampal DG (−2.5~−4.0 mm AP), and every eight sections were stained with antibodies and DAPI (Southern Biotech, USA) and visualized using ZEISS Axioscan 7 (Germany). The positive cells were counted with the “Cell Counter” plugin of the ImageJ software (NIH, US), and the overall number was calculated as cell count per mm^3^ volume of the ventral DG. Antibodies used are listed in Table S1.

### 2.9 Sucrose Preference Test (SPT)

The Sucrose Preference Test (SPT) was conducted following a previous study [6]. After acclimatization to a single cage, the mice were provided with two identical drinking bottles for three consecutive days and given free access to either a 1% sucrose solution or clean water. The volume of the solution in each bottle was measured, and the sucrose preference percentage was calculated as (sucrose solution volume)/(sucrose solution volume + water volume) × 100%.

### 2.10 Open Field Test (OFT)

The test was performed in an acrylic chamber of 40 × 40 × 30 cm^3^, with the bottom divided into 16 equivalent squares, each measuring 10 × 10 cm^2^. The four squares in the center were defined as the central zone, while the other 12 squares were defined as the peripheral zone. Each mouse was carefully placed at the center of the container and allowed for free exploration for 5 min, with the path recorded using a video camera. Time spent in the central and entire areas was analyzed using the ANYmaze 6.0 software (Stoeling, USA). To remove olfactory cues, the chambers were thoroughly cleaned with 75% ethanol and clean water after each test.

### 2.11 Tail Suspension Test (TST)

The tail of the mouse was suspended on a station by adhesive tape approximately 2 cm from the tip for a duration of 6 min when immobility was defined as no initiated movement. The initial 2 min of the adaptation was not recorded, and the following 4 min of remaining motionless were recorded.

### 2.12 Forced Swimming Test (FST)

The mice were placed in clear glass cylinders with a height of 20 cm and a diameter of 14 cm filled with 16 cm deep water at room temperature (25°C ± 1°C), and were allowed to swim freely for 6 min. The initial 2 min was for adaptation, while the next 4 min was recorded for analysis of animal behavior. Despair behavior was defined as immobility except for a single paw flick to remain afloat. The experimental groups were blinded to the investigators.

### 2.13 Target Gene Prediction of miRNAs

The target genes of miRNAs were predicted using the TargetScan (http://www.targetscan.org) and the miRDB (http://www.mirdb.org) databases. Conserved miRNAs were identified by both algorithms, and overlapping candidates were selected when meeting the following criteria: mirSVR score < −0.5, weighted context ++ score < −0.1, and conserved branch length > 3.0. The heatmap was plotted by https://www.bioinformatics.com.cn, an online platform for data analysis and visualization.

### 2.14 Dual‐Luciferase Reporter Assay

The dual luciferase reporter gene was utilized to analyze the binding between miR‐199‐3p and *Mecp2* 3′UTR. The *Mecp2* 3′UTR fragment was amplified by PCR and cloned downstream of a firefly luciferase gene, and the mutated miR‐199‐3p binding site was constructed using the QuickChange site‐directed mutagenesis kit (Stratagene, CA). The luciferase intensity was evaluated using the Dual‐Lumi Luciferase Reporter Gene Assay Kit (Bicentennial, China), and the results were normalized against the *Renilla reniformis* luciferase expressed by pRL‐CMV (Promega).

### 2.15 Statistical Analysis

Data represent means ± SEM of a minimum of four independent experiments. The D'Agostino‐Pearson test was used to determine the normality of data groups with adequate quantities of samples (*n* ≥ 8). For data with normal/Gaussian distribution, unpaired Student's *t*‐tests were used to analyze differences between two groups, while analysis of variance (ANOVA) followed by Newman–Keuls post hoc comparisons were employed for comparing data from three or more groups, depending on the experimental design, using either independent or repeated measurements. Prism 10.0 (GraphPad Software, USA) was used for all statistical analyses and curve fitting. The level of statistical significance was set at *p* < 0.05.

## 3 Results

### 3.1 Chronic Neuropathic Pain Induced Comorbid Depression‐Like Behaviors

**FIGURE 1:**
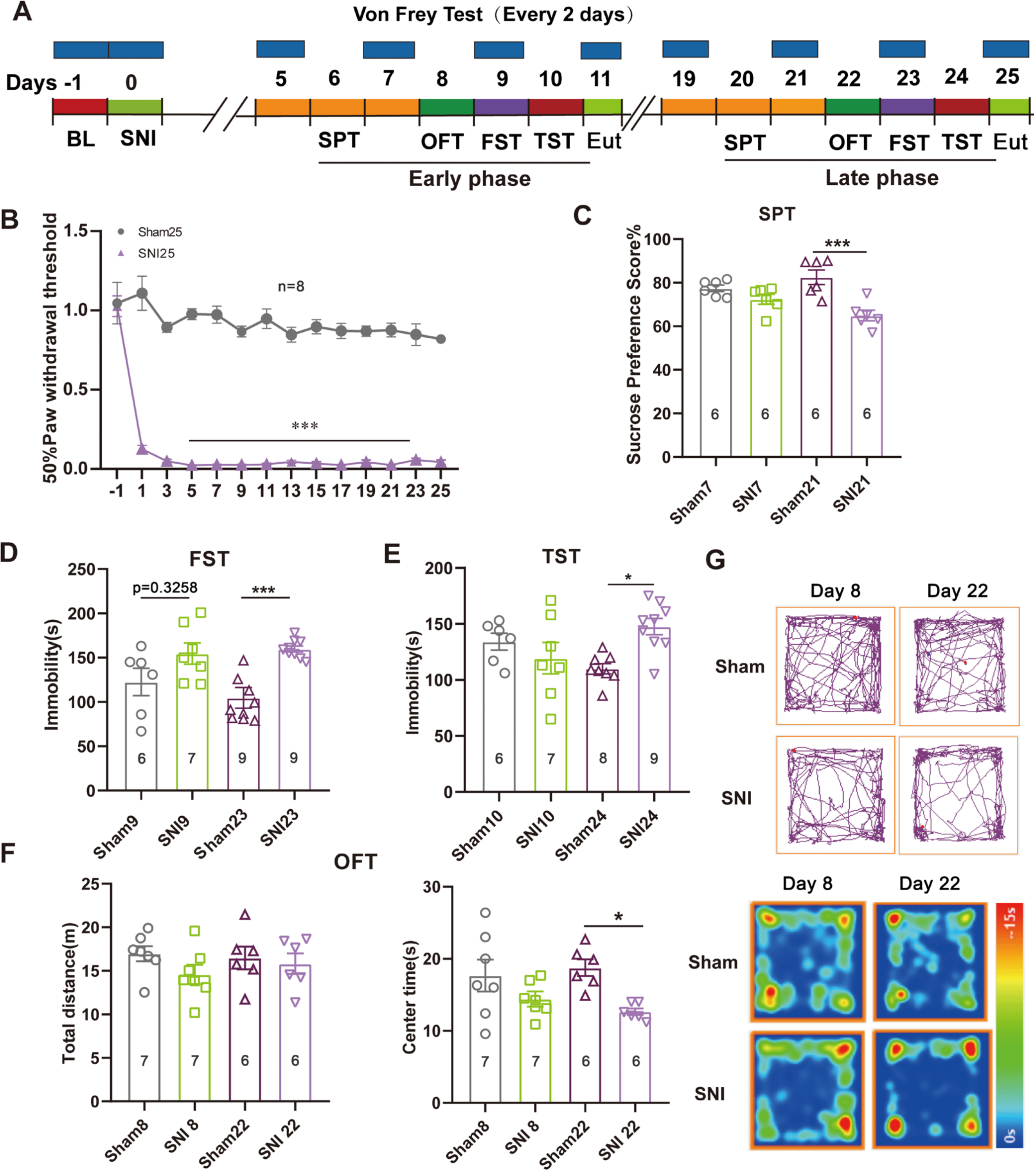
Depression‐like behaviors developed in mice with SNI surgery. (A) Experimental design schedule of the von Frey tests and four behavioral tests (SPT, OFT, FST, and TST) administered at early and late postsurgical phases. BL: Baseline; Eut: Euthanized. (B) The pain threshold tested every 2 days, calculated as 50% PWT. ***p < 0.001 compared with sham; error bars represent SEM; *n* = 8. Statistical analysis was performed using a two‐way ANOVA. (C–E) Performance of mice with sham or SNI surgery in SPT (C), FST (D), and TST (E) on indicated days of the experiment. *p < 0.05, ***p < 0.001 compared with sham; error bars represent SEM. *n* ≥ 6. Statistical analysis was performed using a one‐way ANOVA. (F) Performance of mice with sham or SNI surgery in OFT on indicated days of the experiment, showing the total distance (left panel) and time spent in the central region (right panel). *p < 0.05 compared with sham; error bars represent SEM. *n* ≥ 6. Statistical analysis was performed using a one‐way ANOVA. (G) Representative diagrams of movement tracks and activity heat maps in OFT on indicated days after sham or SNI surgery.

To investigate the effect of chronic neuropathic pain on psychiatric disorders, we prepared the spared nerve injury (SNI) model in mice and assessed the depression‐like behaviors on the 11th and 25th days after surgery (Figure 1A). The von Frey tests were conducted every other day after surgery, showing a stable and significant decrease in mechanical pain threshold in SNI mice compared to those that received sham surgery (Figure 1B). To assess the presence of chronic pain‐induced depression, depression‐like behaviors were evaluated at two distinct time points, either during postsurgical days 5–10 or 19–24, with four behavioral testing paradigms: SPT, OFT, FST, and TST. The depressive‐like behaviors remained undetectable during the early postsurgical phase (days 5–10) (Figure 1D) but became evident in all four behavioral tests during the late postsurgical phase (days 19–24) (Figure 1C–F). In the OFT, although no significant difference in locomotor activity was detected, as indicated by the total distance traveled, there was a significant difference in time spent in the central area on day 22 (Figure 1F,G). These findings suggest the development of depressive‐like behaviors following chronic neuropathic pain. To analyze the impact of gender differences on pain and pain‐related behavior of mice, we compared pain sensitivity and pain‐induced depression in male and female mice, but no significant difference was discovered between the two genders (Figure S1).

### 3.2 Chronic Neuropathic Pain Inhibited Neuronal Differentiation of NSCs in the DG of Adult Mice

**FIGURE 2:**
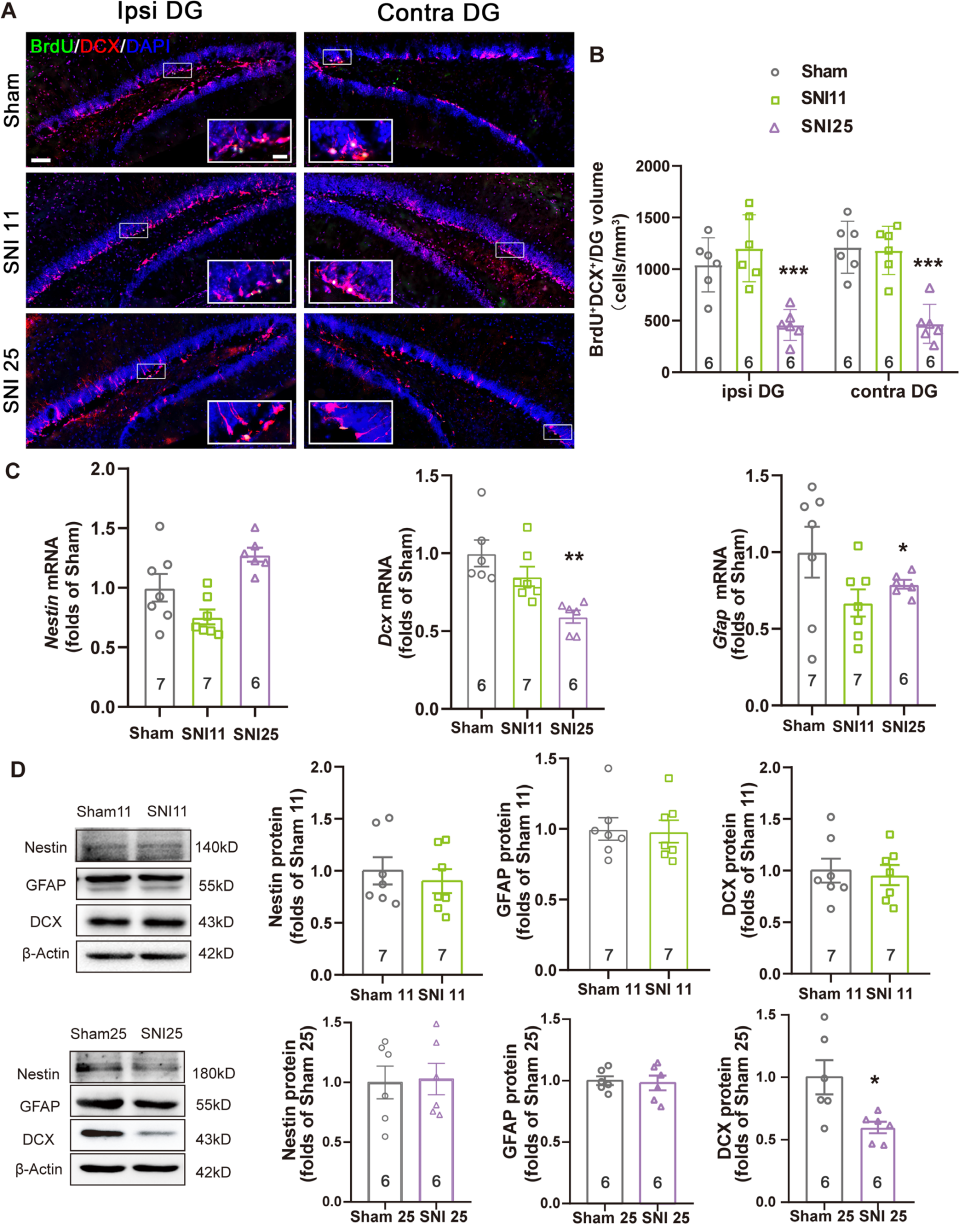
Inhibition of neuronal differentiation in SNI mice with chronic neuropathic pain. (A) Immature neurons (BrdU+DCX+) in the ipsilateral and contralateral ventral DG of mice with sham or SNI surgery on indicated days of the experiment. Images represent six individual animals with similar results. Scale bars: 100 μm for the entire DG and 25 μm for local enlarged panels. (B) The quantification of BrdU+DCX+ immature neurons calculated as the number of positive cells per mm3 volume of the DG on indicated days. Error bars represent SEM. ***p < 0.001 compared with the sham group on the same side; *n* = 6. Statistical analysis was performed using a one‐way ANOVA. (C) The mRNA levels of Nestin, Gfap, and Dcx in the hippocampal ventral DG on indicated days of the experiment, detected by qRT‐PCR. Error bars represent SEM. *p < 0.05; **p < 0.01 compared with the sham group, *n* ≥ 6. Statistical analysis was performed using a one‐way ANOVA. (D) The protein levels of Nestin, GFAP, and DCX in the hippocampal ventral DG on indicated days of the experiment, obtained by western blot, with β‐actin as the internal control. The results were normalized against the sham groups. Error bars represent SEM. *p < 0.05 compared with the sham group, *n* ≥ 6. Statistical analysis was performed using a one‐way ANOVA.

Considering the association between chronic pain‐induced depression and AHN, next we examined adult neurogenesis in the ventral DG using the BrdU labeling assay, on the 11th and 25th days following the SNI surgery. Although no significant difference was observed on postsurgical day 11, a dramatic decrease of the BrdU^+^DCX^+^ cell number was detected on day 25, on both the ipsilateral and the contralateral sides of the ventral DG (Figure 2A,B), suggesting a universal inhibitory effect on neuronal differentiation of NSCs initiated by chronic pain, regardless of the location of the afferent fibers related to nociception. The two other aspects of AHN, proliferation and apoptosis of NSCs in the DG of mice with sham or SNI surgery, were also examined by detecting Ki67^+^ cells (Figure S2) and by using the terminal deoxynucleotidyl transferase dUTP nick labeling (TUNEL) assay (Figure S3, see Supporting Information S1 for the detailed method of TUNEL assay), respectively. As a result, no significant differences were discovered in the number of Ki67^+^ cells or TUNEL^+^ cells, on day 11 or day 25 after the surgery, on either the ipsilateral or the contralateral sides of the DG (Figures S2B and S3B), suggesting that only neuronal differentiation, but not proliferation or survival of NSCs in the ventral DG was affected by chronic neuropathic pain.

The inhibition of neuronal differentiation following chronic pain was further confirmed by examining the mRNA and protein levels related to NSC differentiation in the hippocampal DG of mice with sham or SNI surgery, on days 11 and 25, by using qRT‐PCR (Figure 2C) and western blot assays (Figure 2D). As a result, no significant difference was detected on day 11, while DCX expression in SNI mice was significantly suppressed on day 25, on both the mRNA and protein levels, thus agreeing with the results obtained by the BrdU labeling assay. The protein levels of Nestin, the specific marker of NSCs, and GFAP, the astrocyte marker, remained constant regardless of sham or SNI surgery, though a decrease in *Gfap* transcription was detected. Thus, we may conclude that chronic pain may result in AHN impairment. Furthermore, the association between depression‐like behavior and inhibited AHN induced by chronic pain was indicated by the coincidence in the temporal patterns.

### 3.3 MeCP2 Regulated AHN in Mice With Chronic Neuropathic Pain

**FIGURE 3:**
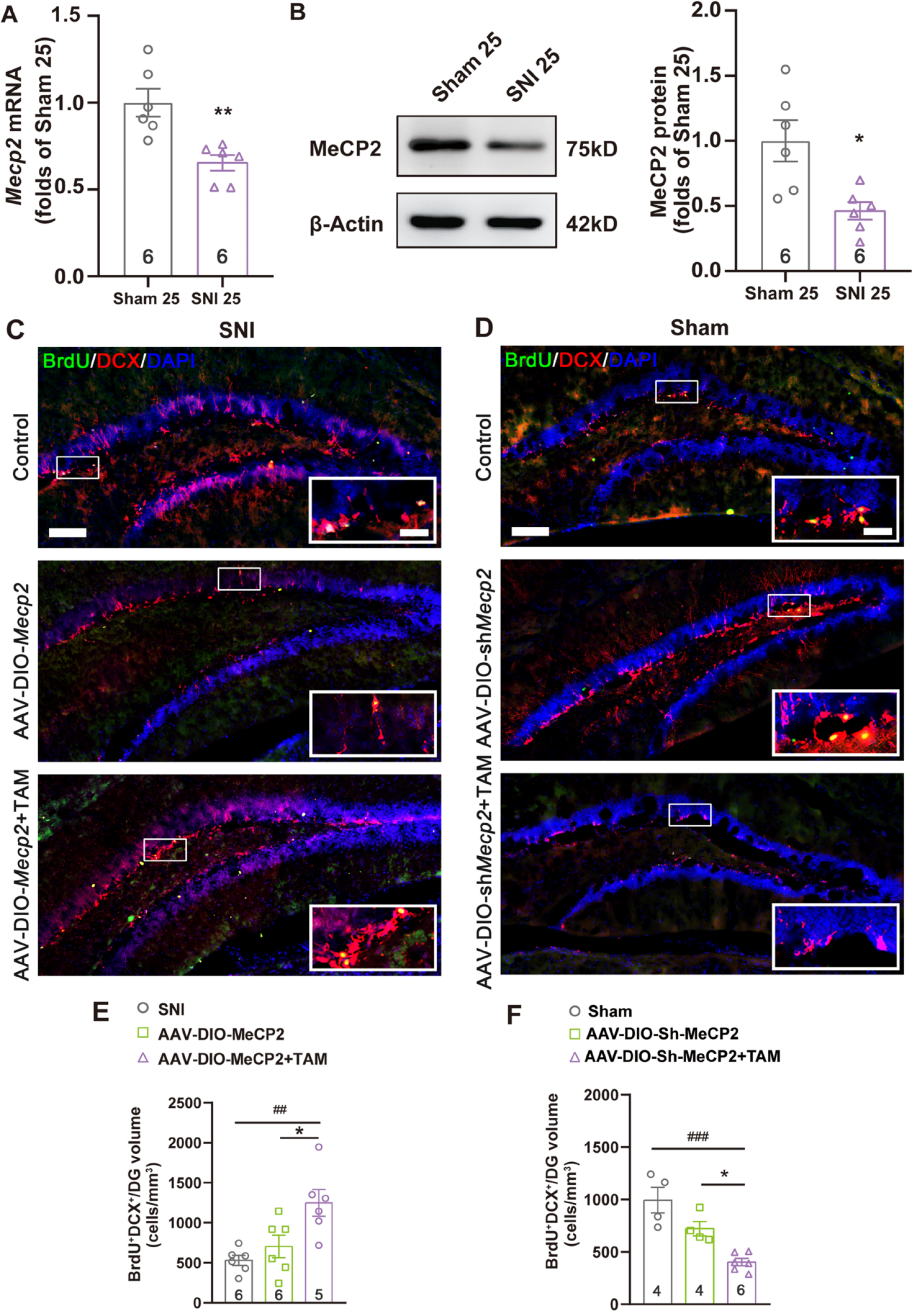
MeCP2 regulated AHN in mice with SNI surgery. (A) The mRNA level of *Mecp2* in the DG of mice with sham or SNI surgery, detected by qRT‐PCR. Error bars represent SEM. **p < 0.01 compared with the sham group, *n* = 6. Statistical analysis was performed using a one‐way ANOVA. (B) The protein level of MeCP2 in the DG of mice with sham or SNI surgery, detected by western blot, with β‐actin as the internal control. The results were normalized against the sham group. Error bars represent SEM. *p < 0.05 compared with the sham group, *n* = 6. Statistical analysis was performed using a one‐way ANOVA. (C, D) Fluorescent images showing the co‐localization of DCX, BrdU, MeCP2, and DAPI after transfection of AAV‐DIO‐*Mecp2* in mice with SNI surgery (C), or the injection of AAV‐DIO‐*Mecp2*shRNA in mice of the sham group (D), with or without tamoxifen administration. Images represent 4–6 individual animals with similar results. Scale bars: 100 μm for the entire DG and 25 μm for local enlarged panels. Statistical analysis was performed using a one‐way ANOVA. (E, F) Quantification of BrdU+DCX+ immature neurons in the ventral DG shown in C and D, respectively, calculated as the number of positive cells per mm^3^ volume. *p < 0.05 compared with the group with AAV injection but without tamoxifen administration; ^##^ *p* < 0.01; ^###^ *p* < 0.001 compared with the group without AAV injection or tamoxifen administration. TAM: Tamoxifen. Error bars represent SEM. *n* = 6. Statistical analysis was performed using a one‐way ANOVA.

Since MeCP2 was proven to participate in AHN regulation [26], we next examined whether the alteration in MeCP2 expression was related to inhibited AHN after SNI surgery. By evaluating MeCP2 expression in the ventral DG of mice experiencing chronic pain using qRT‐PCR (Figure 3A) and western blot (Figure 3B), we revealed a significant reduction in both mRNA and protein levels on the 25th day after SNI surgery, indicating the downregulation of MeCP2 expression in this region of mice with chronic pain and comorbid depression.

To further confirm the role of MeCP2 in the regulation of AHN, we either overexpressed or downregulated MeCP2 expression in hippocampal NSCs by injecting AAVs with the DIO system into the ventral DG of the *Nes*‐CreER^T2^ mice, with tamoxifen administered for five consecutive days to enable the translocation of CreER^T2^ and to initiate the expression of the target genes. As shown in Figure 3C,E, MeCP2 overexpression with AAV‐DIO‐*Mecp2* injection accompanied by tamoxifen administration was able to restore the AHN level in the ventral DG of mice on day 25 after the SNI surgery, as indicated by the number of BrdU^+^DCX^+^ immature neurons. On the contrary, knockdown of MeCP2 in NSCs in the DG with AAV‐DIO‐*Mecp2*shRNA inhibited AHN, characterized by a dramatic decrease of BrdU^+^DCX^+^ cells, thus simulating the effect of chronic neuropathic pain (Figure 3D,F). These results suggested an essential role of MeCP2 in regulating AHN in mice with chronic neuropathic pain.

### 3.4 NSC‐Expressed MeCP2 Regulates Depression‐Like Behaviors of Mice With Chronic Pain

**FIGURE 4:**
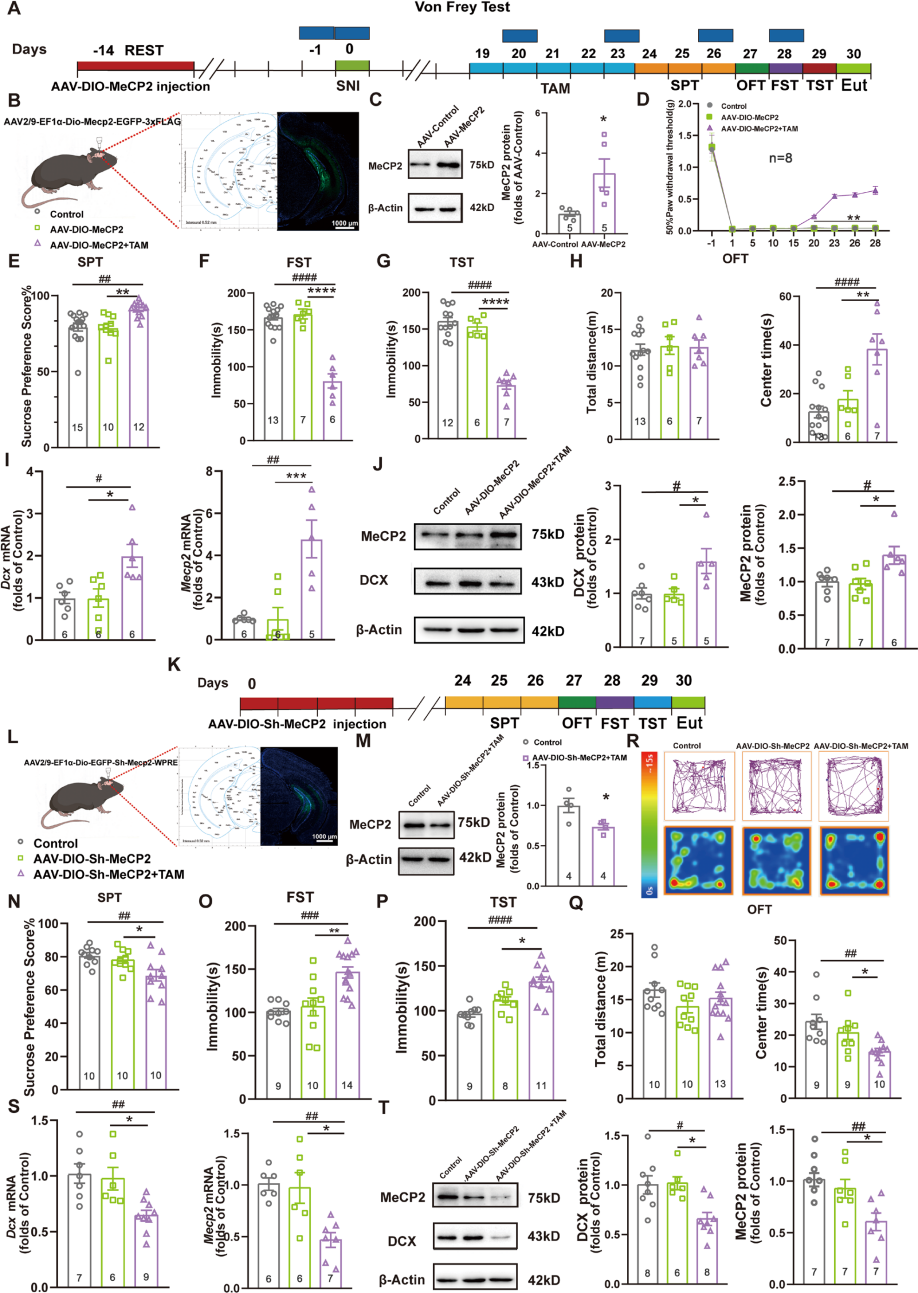
NSC‐expressed MeCP2 regulates depression‐like behaviors of mice with chronic pain. (A, K) The schedule of the experimental design to determine the effect of MeCP2 (A) overexpression and *Mecp2* shRNA (K) on chronic pain comorbid depression of mice with or without SNI surgery. TAM: Tamoxifen; Eut: Euthanized. (B, L) Injection of Cre‐dependent AAV in the ventral DG of mice for the overexpression of MeCP2‐EGFP and *Mecp2*shRNA‐EGFP in hippocampal NSCs. The fluorescent images show the efficiency of AAV‐DIO‐*Mecp2*‐EGFP (B) and AAV‐DIO‐*Mecp2*shRNA‐EGFP (L) transfection. Green: *Mecp2*‐EGFP (B) and *Mecp2*shRNA‐EGFP (L); Blue: DAPI. Scale bar: 1000 μm. (C, M) Immunoblots of the MeCP2 protein after the transfection of the control AAV and AAV‐*Mecp2* (C), or control AAV and AAV‐DIO‐*Mecp2*shRNA (M), with β‐actin as the internal control. The results were normalized against the control group. *p < 0.05 compared with control. Error bars represent SEM. *n* = 6 for C, and *n* = 4 for M. Statistical analysis was performed using a one‐way ANOVA. (D) The pain threshold of mice with injection of indicated AAVs on different days after the SNI surgery. **p < 0.05 compared with the control group and the AAV‐DIO‐*Mecp2* group without tamoxifen administration; *n* = 8. Statistical analysis was performed using a two‐way ANOVA. (E–H & N–Q) Performance of mice with sham or SNI surgery in SPT (E & N), FST (F & O), TST (G & P), and OFT (H & Q) on days 26–29 of the experiment, with the transfection of AAV‐DIO‐*Mecp2* (E–H) or AAV‐DIO‐*Mecp2*shRNA (N–Q). *p < 0.05; **p < 0.01; ****p < 0.0001 compared with the group with AAV transfection but without tamoxifen administration; ^##^ *p* < 0.01; ^###^ *p* < 0.001; ^####^ *p* < 0.0001 compared with the control group without AAV transfection; error bars represent SEM; *n* ≥ 6. Statistical analysis was performed using a one‐way ANOVA. (R) Representative diagrams of movement tracks and activity heat maps in OFT with transfection of indicated AAVs. (I, S) The mRNA levels of *Dcx* and *Mecp2* in the hippocampal ventral DG on day 30 of the experiment, detected by qRT‐PCR. *p < 0.05; ***p < 0.001 compared with the group with AAV transfection but without tamoxifen administration; ^#^ *p* < 0.05; ^##^ *p* < 0.01; ^###^ *p* < 0.001 compared with the control group without AAV transfection; error bars represent SEM; *n* ≥ 5. Statistical analysis was performed using a one‐way ANOVA. (J, T) The protein levels of MeCP2 and DCX in the hippocampal ventral DG on day 30 of the experiment, obtained by western blot, with β‐actin as the internal control. The results were normalized against the control groups. *p < 0.05 compared with the group with AAV transfection but without tamoxifen administration; ^#^ *p* < 0.05; ^##^ *p* < 0.01 compared with the control group without AAV transfection; error bars represent SEM; *n* ≥ 5. Statistical analysis was performed using a one‐way ANOVA.

Now that we have proved that AHN was related to chronic pain–induced depression and that MeCP2 was crucial in AHN regulation, we next investigated the role of MeCP2 in the depression‐like behavior of mice after SNI surgery. By selectively increasing MeCP2 expression in hippocampal NSCs of *Nes*‐CreER^T2^ mice with AAV injection 14 days prior to the SNI surgery (Figure 4A), we investigated the effect of MeCP2 on both pain sensitivity and pain‐related emotion. The efficiency of the AAV was tested by injecting AAV‐*Mecp2* into the mouse hippocampus (Figure 4B), which resulted in a significantly increased protein level of MeCP2 (Figure 4C). By using the von Frey filament assay, we evaluated pain sensitivity in mice with and without MeCP2 expression and revealed decreased pain sensitivity upon MeCP2 overexpression (Figure 4D). Behavioral tests including the SPT, OFT, FST, and TST were conducted to assess the depression‐like behaviors in mice experiencing chronic pain, demonstrating that MeCP2 overexpression in hippocampal NSCs was able to alleviate the depression‐like behaviors of these mice (Figure 4E–H). By examining the mRNA and protein levels via the qRT‐PCR and western blot assays, we were able to detect significant upregulated expression of both MeCP2 and DCX (Figure 4I,J), indicating the connection between MeCP2 expression, AHN, and depression‐like behaviors.

On the other hand, knockdown of *Mecp2* was performed in normal mice without SNI (Figure 4K) by using an AAV construct that specifically expressed *Mecp2* shRNA in the NSCs of *Nes*‐CreER^T2^ mice (Figure 4L), resulting in a significant decrease in MeCP2 expression (Figure 4M). The behavior tests demonstrated remarkable depression‐like symptoms after *Mecp2* knockdown (Figure 4N–R), accompanied by downregulated expression of MeCP2 and DCX on both the mRNA and protein levels (Figure 4S,T). Thus, the results above further confirmed the crucial role of MeCP2 in modulating AHN and depression‐like behavior induced by chronic neuropathic pain. However, the mechanisms that control MeCP2 expression in hippocampal NSCs of mice with chronic pain remained elusive.

### 3.5 Analyses of miRNA Expression Profiles in the Ventral DG of Mice With Chronic Pain–Induced Depression

**FIGURE 5:**
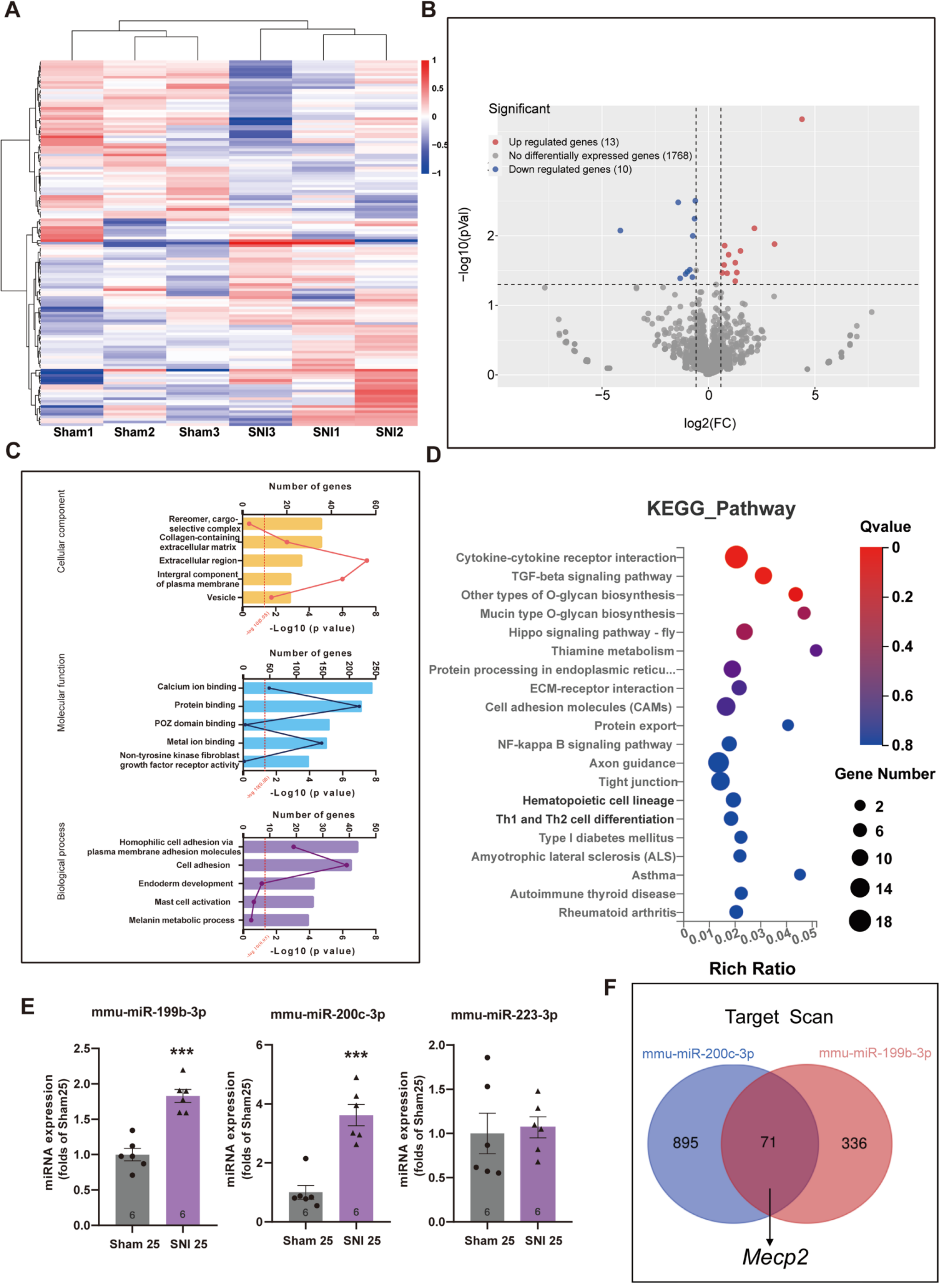
MiRNA analyses in the ventral DG of mice with chronic neuropathic pain. (A, B) The heatmap and volcano diagrams of miRNAs expressed in the ventral DG of mice, 25 days after the sham or SNI surgeries. *n* = 3. Statistical analysis was performed using a one‐way ANOVA. (C) Functional analysis of the DEGs via GO analysis, showing the annotated functions of target genes, along with the enrichment score (−log10 transformed *p*‐values) reflecting the number of genes in each cluster. (D) The KEGG pathway enrichment analysis of DEGs, with the color of bubbles corresponding to the q values, and the area of bubbles representing the number of genes. (E) Confirmation of the miRNA candidates using the qRT‐PCR assay. ***p < 0.001 compared with the sham group; error bars represent SEM; *n* = 6. Statistical analysis was performed using a one‐way ANOVA. (F) Prediction of the target genes of the two candidate miRNAs by analyzing the overlapping of the TargetScan and miRDB databases, showing Mecp2 as a potential target of both miRNAs.

As the collaboration between miRNAs and MeCP2 has been recognized as a common paradigm in AHN regulation [26, 30], we analyzed miRNA expression patterns in the ventral DG of mice with chronic pain comorbid with depression by miRNA sequencing. Results from cluster analysis demonstrated high uniformity between the SNI and sham groups, and marked differences in gene expression between the two groups were detected (Figure 5A). Based on the criteria of *p* < 0.05 and |log2 Fold Change| > 1, we identified 23 differentially expressed genes (DEGs), including 13 upregulated and 10 downregulated genes (Figure 5B). The DEGs were used for gene ontology (GO) analysis regarding their enrichment scores in terms of biological processes, cellular components, and molecular functions (Figure 5C). Moreover, the KEGG pathway enrichment analysis was used to analyze the pathways regulated by the candidate miRNAs (Figure 5D).

Among the significantly differentially expressed genes, we selected three miRNAs that were found to be involved in the regulation of neural functions or stem cells: mmu‐miR‐199b‐3p [31], mmu‐miR‐200c‐3p [32], and mmu‐miR‐223‐3p [33] were selected, and their expressions were verified by qRT‐PCR. Both miR‐199b‐3p and miR‐200c‐3p were prominently upregulated in the DG of mice with chronic pain, whereas miR‐223‐3p showed no significant difference in the two groups (Figure 5E). Thus, these two miRNAs were selected as the remaining candidates that may regulate MeCP2 expression and were subjected to miRNA target prediction using two databases, targetscan and miRDB, both supporting that *Mecp2* may be a potential target of the two miRNAs (Figure 5F).

### 3.6 MeCP2 Is a Target of miR‐199b‐3p

**FIGURE 6:**
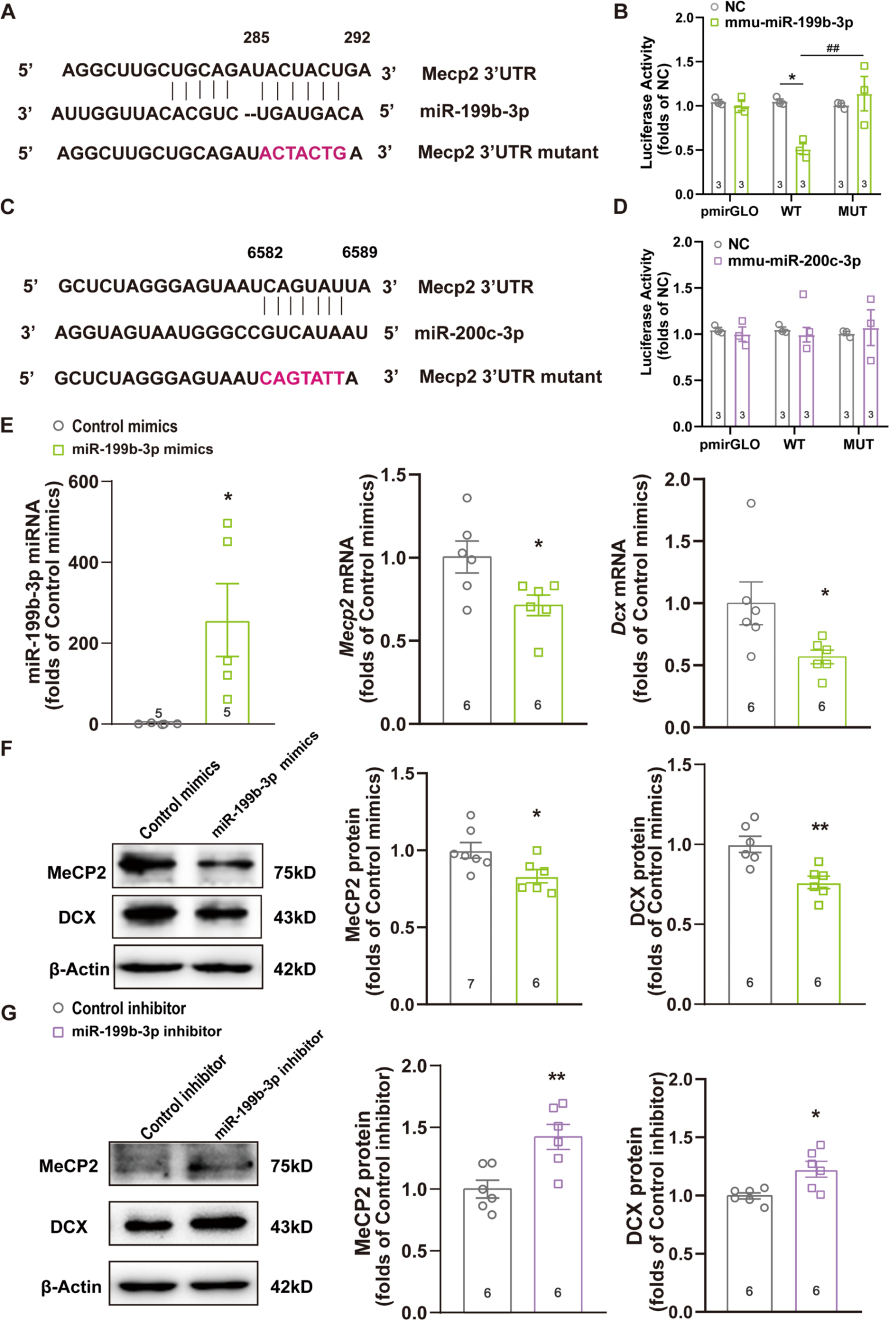
Mecp2 is a Target of miR‐199b‐3p. (A, C) Schematics of the wildtype and mutant *Mecp* 3′UTR reporters, with the predicted conserved binding site for miR‐199b‐3p (A) and miR‐200c‐3p (C). The mutated sequences were marked as pink‐colored. (B, D) The luciferase activities after the cotransfection of miR‐199b‐3p (B) or miR‐200c‐3p (D), along with the wildtype and mutant *Mecp* 3′UTR fragments. *p < 0.05 compared with the negative control (NC); ^##^ *p* < 0.01 compared between the wildtype (WT) and the mutant (MUT) *Mecp* 3′UTR. Error bars represent SEM; *n* = 3. Statistical analysis was performed using a one‐way ANOVA. (E) The miRNA levels of miR‐199b‐3p and the mRNA levels of *Mecp*2 and *Dcx* after transfection with the miR‐199b‐3p mimic. *p < 0.05; error bars represent SEM; *n* ≥ 5. Statistical analysis was performed using a one‐way ANOVA. (F, G) The effect of the mimic (F) or inhibitor (G) of miR‐199b‐3p on MeCP2 and DCX protein levels detected by western blotting, with β‐actin used as an internal control. *p < 0.05; **p < 0.01 compared with the control mimics group; error bars represent SEM; *n* ≥ 6. Statistical analysis was performed using a one‐way ANOVA.

To confirm the association between *Mecp2* 3′ UTR and the two candidate miRNAs, we conducted a dual‐luciferase reporter assay in HEK293T cells by cotransfecting either miR‐199b‐3p or miR‐200c‐3p subcloned into the pmirGLO vector, along with the pGL3 luciferase reporter vector containing the wildtype or mutant *Mecp2* 3′ UTR, the mutation of which was developed at the targeting site of each miRNA (Figure 6A–D). As a result, the relative luciferase activity significantly decreased in cells cotransfected with the wild‐type *Mecp2* 3′UTR plasmid and miR‐199b‐3p, compared to the negative control (NC) of miR‐199b‐3p. However, such inhibition of luciferase activity was absent when the mutant *Mecp2* 3′ UTR was transfected, indicating a direct association between *Mecp2* 3′ UTR and miR‐199b‐3p (Figure 6B). On the other hand, in cells cotransfected with *the Mecp2 3*′ *UTR* plasmid and miR‐200c‐3p, the luciferase activity remained constant, regardless of the wildtype or mutant sequences (Figure 6D), thus excluding the involvement of miR‐200c‐3p in MeCP2 regulation.

To further elucidate the regulation of MeCP2 and AHN by miR‐199b‐3p, we examined the expression of MeCP2 and DCX in primary NSCs after transfection of the miR‐199b‐3p mimic. As shown in Figure 6E, the mimic of miR‐199b‐3p, which was able to dramatically enhance the miRNA function, led to significantly lower mRNA levels of *Mecp2* and *Dcx*, with the simultaneous downregulation of their protein levels (Figure 6F). On the contrary, transfection with the miR‐199b‐3p inhibitor resulted in significantly increased protein levels of MeCP2 and DCX (Figure 6G). Such inverse regulation confirmed that miR‐199b‐3p inhibited MeCP2 expression by directly targeting and binding to 3′ UTR of *Mecp2*, which in turn modulated AHN by controlling the neuronal differentiation of hippocampal NSCs.

### 3.7 Knockdown of miR‐199b‐3p Restored AHN and Alleviated Chronic Pain Comorbid Depression by Increasing MeCP2 Expression

**FIGURE 7:**
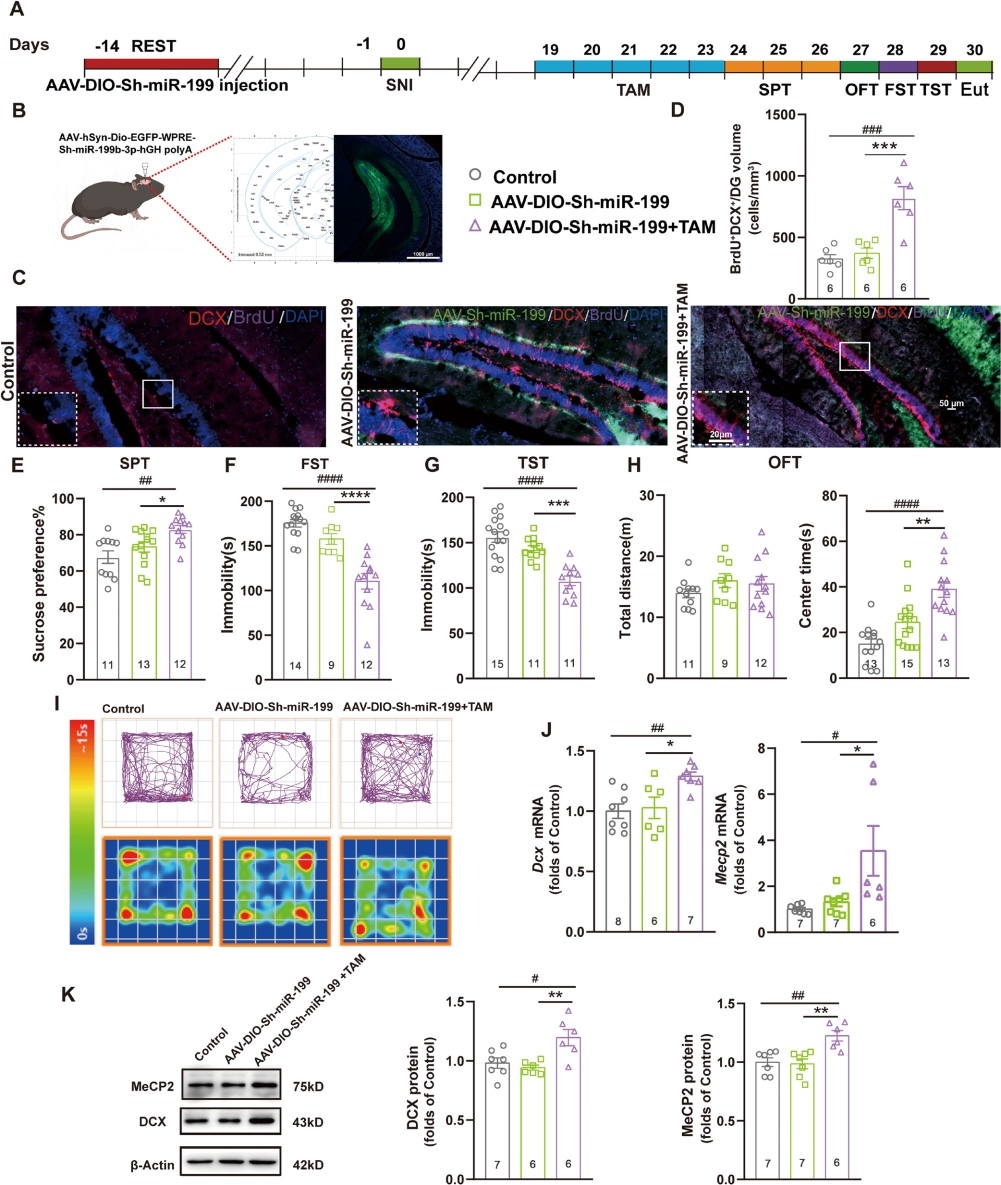
MiR‐199b‐3p regulated AHN and depression‐like behavior in mice with chronic pain. (A) The schedule of the experimental design to determine the effect of AAV‐DIO‐sh‐miR‐199 transfection on AHN and depression‐like behavior of mice with SNI surgery. TAM: Tamoxifen; Eut: Euthanized. (B) Injection of Cre‐dependent AAV in the ventral DG of mice for the knockdown of miR‐199b‐3p in hippocampal NSCs. The fluorescent images show the efficiency of AAV‐DIO‐sh‐miR‐199‐EGFP transfection. Green: Sh‐miR‐199‐EGFP; Blue: DAPI. Scale bar: 1000 μm. (C) Fluorescent images showing the co‐localization of sh‐miR‐199‐EGFP, DCX, BrdU, and DAPI after transfection of AAV‐DIO‐sh‐miR‐199‐EGFP in mice, with or without tamoxifen administration. Images represent six individual animals with similar results. Scale bars: 50 μm for the entire DG and 20 μm for local enlarged panels. (D) Quantification of BrdU+DCX+ immature neurons in the ventral DG shown in (C), calculated as the number of positive cells per mm3 volume. ***p < 0.001 compared with the group with AAV injection but without tamoxifen administration; ^###^ *p* < 0.001 compared with the group without AAV injection or tamoxifen administration. TAM: Tamoxifen. Error bars represent SEM. *n* = 6. Statistical analysis was performed using a one‐way ANOVA. (E–H) Performance of mice with SNI surgery and AAV‐DIO‐sh‐miR‐199 transfection in SPT (E), FST (F), TST (G), and OFT (H) on days 26–29 of the experiment. *p < 0.05; **p < 0.01; ***p < 0.001; ****p < 0.0001 compared with the group with AAV transfection but without tamoxifen administration; ^##^ *p* < 0.01; ^####^ *p* < 0.0001 compared with the control group without AAV transfection; error bars represent SEM; *n* ≥ 9. Statistical analysis was performed using a one‐way ANOVA. (I) Representative diagrams of movement tracks and activity heat maps in OFT with transfection of indicated AAVs. (J) The mRNA levels of *Dcx* and *Mecp2* in the hippocampal ventral DG on day 30 of the experiment, detected by qRT‐PCR. *p < 0.05 compared with the group with AAV transfection but without tamoxifen administration; ^#^ *p* < 0.05; ^##^ *p* < 0.01 compared with the control group without AAV transfection; error bars represent SEM; *n* ≥ 6. Statistical analysis was performed using a one‐way ANOVA. (K, L) The protein levels of MeCP2 and DCX in the hippocampal ventral DG on day 30 of the experiment, obtained by western blot, with β‐actin as the internal control. The results were normalized against the control groups. **p < 0.01 compared with the group with AAV transfection but without tamoxifen administration; ^#^ *p* < 0.05; ^##^ *p* < 0.01 compared with the control group without AAV transfection; error bars represent SEM; *n* ≥ 6. Statistical analysis was performed using a one‐way ANOVA.

Now that we have proved that MeCP2 regulated AHN and depression in mice with chronic neuropathic pain and confirmed the role of miR‐199b‐3p in targeting and inhibiting MeCP2 expression, we next examined the effect of miR‐199b‐3p on AHN and depression‐like behaviors of mice with SNI surgery. The ventral DG of *Nes*‐CreER^T2^ mice was injected with AAV‐DIO‐sh‐miR‐199 14 days before SNI surgery (Figure 7A), which enabled the knockdown of miR‐199‐3p in NSCs (Figure 7B). The newborn immature neurons were detected by BrdU labeling and immunofluorescence (Figure 7C), showing a significant increase in the number of BrdU^+^DCX^+^ cells upon the transfection of AAV‐DIO‐sh‐miR‐199, along with tamoxifen administration (Figure 7D), suggesting that the increased activity of miR‐199b‐3p after SNI surgery was an initial factor leading to AHN inhibition. Depressive‐like behaviors were then assessed through the four behavioral paradigms, which revealed alleviated depressive‐like behaviors after the knockdown of miR‐199b‐3p in mice with chronic pain (Figure 7E–I), suggesting the pro‐depressant role of miR‐199b‐3p in hippocampal NSCs of mice with chronic pain. To further confirm the effect of miR‐199b‐3p on the expression of MeCP2 and DCX in the ventral DG, with or without the injection of AAV‐Sh‐miR‐199b‐3p and the administration of tamoxifen, we tested the mRNA and protein levels of both MeCP2 and DCX by using qRT‐PCR and western blot assays, and found significantly upregulated expression of MeCP2 and DCX after the knockdown of miR‐199b‐3p (Figure 7J,K), indicating the role of miR‐199b‐3p in suppressing AHN by inhibiting MeCP2 expression. These results elucidated the mechanism by which miR‐199b‐3p inhibited AHN and induced chronic pain comorbid depression by targeting *MeCP2* in the ventral DG NSCs of mice with SNI surgery.

## 4 Discussion

The present study focused on the mechanisms controlling depression induced by chronic neuropathic pain. By using the *Nestin*‐CreER^T2^ mice and AAV‐induced modification of essential genes, we confirmed the association between AHN and psychiatric disorders, along with the molecular mechanisms around MeCP2, the inhibition of which initiated by miR‐199b‐3p was revealed as an indispensable step that led to AHN inhibition and the occurrence of depression‐like behaviors. The connection between AHN and negative emotions supported by our current work has been proven by numerous studies during the past decade. Despite the similar conclusions demonstrating that AHN inhibition was discovered in animals with depression [6, 34] or anxiety [35], diverse mechanisms were reported, including transcriptional and epigenetic [6] regulation of local molecules in the NSCs, and long‐range control by other brain areas via neural circuits [35], indicating that the MeCP2‐based mechanism might not be unique in regulating AHN and chronic pain comorbid depression. On the other hand, due to the ubiquitous epigenetic functions of MeCP2 and the widely validated association between AHN and psychiatric disorders, we may infer that this miRNA/MeCP2/AHN module discovered in this study might exemplify a common mechanism underlying various psychiatric disorders, working in a way dependent on the interaction with other mechanisms that collaborate in the regulation of psychiatric status in a more complex manner, which is worth further investigation.

In the current work, we found no significant difference in either pain sensitivity or pain‐induced depression between male and female mice, which is consistent with our previous study [6], but conflicts with multiple earlier studies suggesting that females are more sensitive to pain [36, 37]. We believe that two major reasons might give rise to such a discrepancy. First, the gender difference in pain sensitivity may depend on the pain modalities. For example, females might be more sensitive to pressure and thermal pain, but not to ischemic or neuropathic pain [38]. Second, the high intensity of pain sensation may render the existing gender difference insignificant, and therefore more difficult to detect. Overall, the gender difference in pain sensitivity is a complex issue controlled by various factors, which need further studies in the future.

The miRNA/MeCP2 interaction has been revealed in a series of studies during the past decade, with diverse patterns in the regulation of adult neurogenesis. First, MeCP2 could perform as a normal target of miRNAs, which served as upstream regulators of MeCP2‐dependent signaling pathways that modulated neural functions, as was confirmed by the current study. Second, as a transcriptional modulator, MeCP2 was able to regulate the transcription of miRNAs, thus indirectly modulating the expression of miRNA targets, the roles of which in neurogenesis were revealed previously. For example, MeCP2 suppressed miR‐124a processing by blocking the access of Drosha‐DGCR8, thereby restricting neurogenesis [26]. Finally, the activity of MeCP2 relied not only on its expression but also on the phosphorylation status at the Ser421 residue, which determined the nuclear processing of miR‐101a and, in turn, regulated opioid‐induced synaptic and behavioral plasticity via a twinfilin1‐dependent mechanism [23]. These results indicate multiple pathways supporting the functioning of the miRNA/MeCP2 module. Though we have confirmed that miR‐199b‐3p could target MeCP2 in our study, we cannot rule out the probability that other candidate miRNAs, though not targeted by miRNAs, might regulate the processing of certain miRNAs associated with adult neurogenesis. Moreover, MeCP2 phosphorylation should be examined in the future for further exploration of neurogenesis‐related mechanisms.

Although the depression‐like behavior was derived from chronic neuropathic pain, the association between pain perception and pain‐related emotion remains elusive in the present study, as MeCP2 overexpression in the hippocampal NSCs resulted in both increased pain threshold (Figure 4D) and alleviated depression‐like behaviors (Figure 4E–H). It is, thus, difficult to elucidate whether the antidepressant effect of MeCP2 was due to restored AHN or merely a result of alleviated nociception. According to the previous studies of our and other groups, AHN had completely different effects on perceptional and emotional aspects of chronic pain, since ablation of AHN resulted in deterioration of negative emotions, but not pain perception [6, 39]. On the other hand, studies focusing on the functions of MeCP2 confirmed its role in the modulation of both nociception and negative emotion, since MeCP2 was a key regulator not only in pain transgenerational transmission [40, 41], but also in negative emotions [42], thus coinciding with our present study. Considering its ubiquitous effects in controlling gene transfection, as well as the widely discovered targets of MeCP2, it is reasonable to infer that MeCP2 might simultaneously regulate both pain perception and pain‐related depression, but with distinct mechanisms. While we elucidated the mechanisms by which miR‐199b‐3p/MeCP2/AHN controlled chronic pain comorbid depression in the current study, we admit the existence of another MeCP2‐mediated pathway that might lead to the regulation of nociception, which should be clarified in our future studies.

Given the neuroinflammation‐related signaling pathway identified in KEGG analysis of the present study, such as cytokine‐cytokine receptor interaction (Figure 5D), we may infer the involvement of neuroinflammation in SNI‐induced AHN inhibition and depression. According to recent studies, chronic pain‐induced neuroinflammation may impair adult neurogenesis via two major mechanisms. First, chemokines and their receptors expressed on hippocampal NSCs may collaborate in AHN regulation. For example, a high level of C‐X‐C motif chemokine receptor 2 (CXCR2) expression found in mouse DG was associated with NSC proliferation [43]. Moreover, C‐X‐C motif chemokine ligand 1 (CXCL1) was able to activate CXCR2 and improved the survival rate of hippocampal neurons [44]. Second, chronic neuropathic pain initiated by SNI surgery may result in the activation and polarization of microglia [45], which may, in turn, contribute to AHN impairment and give rise to depression‐like behaviors [46]. Therefore, the association between neuroinflammation and AHN impairment is worth further investigation in the exploration of the underlying mechanisms involved in chronic pain comorbid with negative emotions.

Although we have proven that MeCP2 expressed in hippocampal NSCs was responsible for AHN modulation, the detailed mechanisms underlying such effect remain unsolved. We may nevertheless speculate on the downstream pathways regulated by MeCP2, which might directly control AHN. For example, Notch, a critical regulator of NSC differentiation and AHN [14], was found to be directly regulated by MeCP2 [47]. Other studies have shown that activity‐dependent changes in DNA methylation are linked to a decrease in MeCP2 binding on the promoter of the brain‐derived neurotrophic factor (BDNF) [48], which controls AHN through tropomyosin receptor kinase B (TrkB) [49]. Besides, MeCP2, which was targeted by miR‐212‐3p in a manner similar to that discovered in the present work, was able to regulate AHN through the AKT/mTOR signaling pathway [30]. Thus, we may infer that MeCP2 might modulate AHN through pathways mediated by Notch, BDNF, or protein kinases such as AKT in mice with chronic pain comorbid depression. Further investigation is needed for clarification.

The mechanisms that promoted miR‐199b‐3p expression and maturation in mice with chronic pain are another issue that needs further elucidation. We may infer that the transcription of its precursor, the stem‐loop mmu‐mir‐199b, might be altered by certain transcription factors that bind to the promoter under the condition of chronic neuropathic pain. Moreover, since the DNA fragment encoding mir‐199b is located within the intron sequence of its host gene, *Dnm1*, on Chromosome 2, we may speculate that the transcription of *Dnm1* might also be involved in the regulation of chronic pain comorbid depression. As the protein product of *Dnm1*, Dynamin 1, is a GTPase required for membrane recycling and vesicle endocytosis in neurons [50], we hypothesize that altered neurogenesis may involve dynamin‐mediated processes such as vesicle transportation and mitochondrial biogenesis, though more evidence is needed to support this hypothesis.

As a specific method that measures the relative preference of sucrose over clear water, the SPT is considered unique in assessing the hedonic behaviors rather than the motivational efforts of rodents [51]. Since anhedonia may reflect a diverse array of deficits in hedonic functions, encompassing all aspects of the reward system, it may be considered not only a core diagnostic symptom of depression but also an index of the reward circuitry functions [51, 52]. In our current study, miR‐199b knockdown or MeCP2 overexpression in hippocampal NSCs diminished anhedonia in mice with chronic pain, whereas MeCP2 knockdown may give rise to anhedonia in healthy animals (Figure 4). These observations may be attributed to changes in the AHN level upon the alteration of miR‐199b/MeCP2 signaling, agreeing with the previous study proving the correlation between AHN and anhedonia [53]. Besides, the dopaminergic reward circuitry might also participate in such effects. In contrast, the compulsion‐like behavior, reflected by increased sucrose consumption by healthy animals, was not tested in the present study. The SPT using healthy mice with overexpressed MeCP2 or enhanced AHN should be carried out in the future to evaluate the relationship between compulsion and AHN, along with the underlying mechanisms.

The findings of the present study implicate the potential application in clinical practice. Owing to the involvement of the MeCP2 expressed in hippocampal NSCs, it should be considered a molecular target for the treatment of chronic pain comorbid depression. Two different strategies may be adopted for the specific control of NSC‐expressing MeCP2. First, a targeting drug delivery system carrying regulators of MeCP2 may be developed for targeting NSCs in the DG. Second, exosomes carrying a lower concentration of miR‐199b‐3p may be transplanted to the DG of patients with chronic pain‐induced depression. With these methods, we may expect effective treatment of negative emotions derived from chronic pain.

So far, we have elucidated the central role of MeCP2 in regulating depression induced by chronic pain in the present study, due to its ability to regulate gene expression on a posttranscriptional level. Though a series of questions regarding the detailed mechanisms remain unclear, the current work may provide new insights into the putative common mechanism that might be associated with AHN and negative emotions. Thus, further investigation should be focused on the role of MeCP2 in psychiatric and cognitive dysfunctions related to impaired AHN, and MeCP2 should be considered a new target in the treatment of such disorders.

## 5 Conclusion

We demonstrate that chronic neuropathic pain may result in an increased level of miR‐199b‐3p in hippocampal NSCs, which, in turn, targets the *Mecp2* gene and inhibits its transcription. Since normal MeCP2 expression is essential for neuronal differentiation of NSCs, its downregulation may lead to inhibited AHN, which finally causes depression‐like behaviors. Thus, restoration of MeCP2 expression is essential for the alleviation of chronic pain comorbid depression.

## Author Contributions

Yanting Sun was involved in the collection and/or assembly of data, data analysis and interpretation, and manuscript writing. Ying Zhang contributed to the collection and/or assembly of data, data analysis, and interpretation. Yexiang Chen was involved in the collection and/or assembly of data, data analysis, and interpretation. Huisheng Peng contributed to the collection and/or assembly of data. Tiantian Cheng was involved in data analysis and interpretation. Xiujian Sun contributed to the conception and design. Jing‐Gen Liu: contributed to conception and design, financial support, administrative support, and final approval of the manuscript. Chi Xu contributed to the conception and design, financial support, administrative support, data analysis and interpretation, manuscript writing, and final approval of manuscript.

## Conflicts of Interest

The authors declare no conflicts of interest.

## Supporting information


**Data S1** Supplementary methods.

**Figure S1** Chronic pain sensation and comorbid depression‐like behaviors of male and female mice. (A) The pain threshold tested on indicated days after the surgery, calculated as 50% PWT. Error bars represent SEM, *n* = 6. (B–F) Performance of mice with sham or SNI surgery in SPT (B), FST (C), TST (D), and OFT (E, F) on days 21–24 after the surgery. *p < 0.05; **p < 0.01; ***p < 0.001 versus sham male mice. ^#^ *p* < 0.05, ^##^ *p* < 0.01 vs. sham female mice. Error bars represent SEM, *n* = 6. Statistical analysis was performed using a two‐way ANOVA.

**Figure S2** Effects of SNI surgery on the proliferation of hippocampal NSCs. (A) The ipsilateral and contralateral ventral DG of mice with or without SNI surgery were examined by using antibodies against Ki67, the index for cell proliferation, on indicated days. Red: Ki67; Blue: DAPI. Images represent at least six individual animals with similar results. Scale bar: 100 μm. (B) Quantification of Ki67^+^ cells in the ventral DG. Error bars represent SEM, *n* ≥ 6. Statistical analysis was performed using a one‐way ANOVA.

**Figure S3** Effects of SNI surgery on the apoptosis of newborn neurons in the ventral DG. (A) Cell apoptosis in the ipsilateral and contralateral ventral DG of mice with or without SNI surgery on indicated days was examined by TUNEL assay. Red: Ki67; Green: TUNEL; Blue: DAPI. Images represent at least six individual animals with similar results. Scale bar: 100 μm. (B) Quantification of cell apoptosis by comparing the average fluorescence of TUNEL and NeuN, calculated as the TUNEL/NeuN ratio. Error bars represent SEM, *n* ≥ 6. Statistical analysis was performed using a one‐way ANOVA.

**Table S1** Antibodies.

## Data Availability

The data that support the findings of this study are available from the corresponding author upon reasonable request.
