# Supplementary material for: MeCP2 Modulates Depression‐Like Behaviors Comorbid to Chronic Pain by Regulating Adult Hippocampal Neurogenesis
Source: CNS Neurosci Ther. 2025 Apr 7;31(3):e70311. doi: 10.1111/cns.70311 (PMC11974449; doi:10.1111/cns.70311)
Supplement: Supplementary file 1 — Data S1 Supplementary methods. [file CNS-31-e70311-s003.docx]

**Supplementary Methods**

**Terminal deoxynucleotidyl transferase-dUTP nick end labeling (TUNEL) assay**

The TUNEL assay was performed by using the Click-iT® Plus TUNEL Assay kit for in situ apoptosis detection with Alexa Fluor® dyes (ThermoFisher, USA). The mouse brain sections were permeabilized with Proteinase K solution for 15 min, and washed with PBS for 10 min. For positive control, DNA strands are broken by incubating fixed and permeabilized cells with 1 unit of DNase I diluted into 1× DNase I reaction buffer (20 mM Tris-HCl, pH 8.4, 2 mM MgCl2, 50 mM KCl) for 30 min at room temperature. The terminal deoxynucleotidyl transferase (TdT) reaction was performed by adding 100 μl of TdT reaction mixture (containing TdT reaction buffer, EdUTP and TdT enzyme) to each slide and incubating for 1 h at 37°C. The Click-iT® Plus reaction was performed by incubating each slide with 100 μl of Click-iT® Plus reaction cocktail for 30 min at 37°C. After removing the reaction cocktail and washing each coverslip with 3% BSA in PBS for 5 min, cells were mounted on slides with DAPI Fluormount G (SouthernBiotech, USA) and visualized using the ZEISS Axioscan 7 digital slide scanner (Germany).
