# Supplementary figures and images for: MeCP2 Modulates Depression‐Like Behaviors Comorbid to Chronic Pain by Regulating Adult Hippocampal Neurogenesis
Source: CNS Neurosci Ther. 2025 Apr 7;31(3):e70311. doi: 10.1111/cns.70311 (PMC11974449; doi:10.1111/cns.70311)

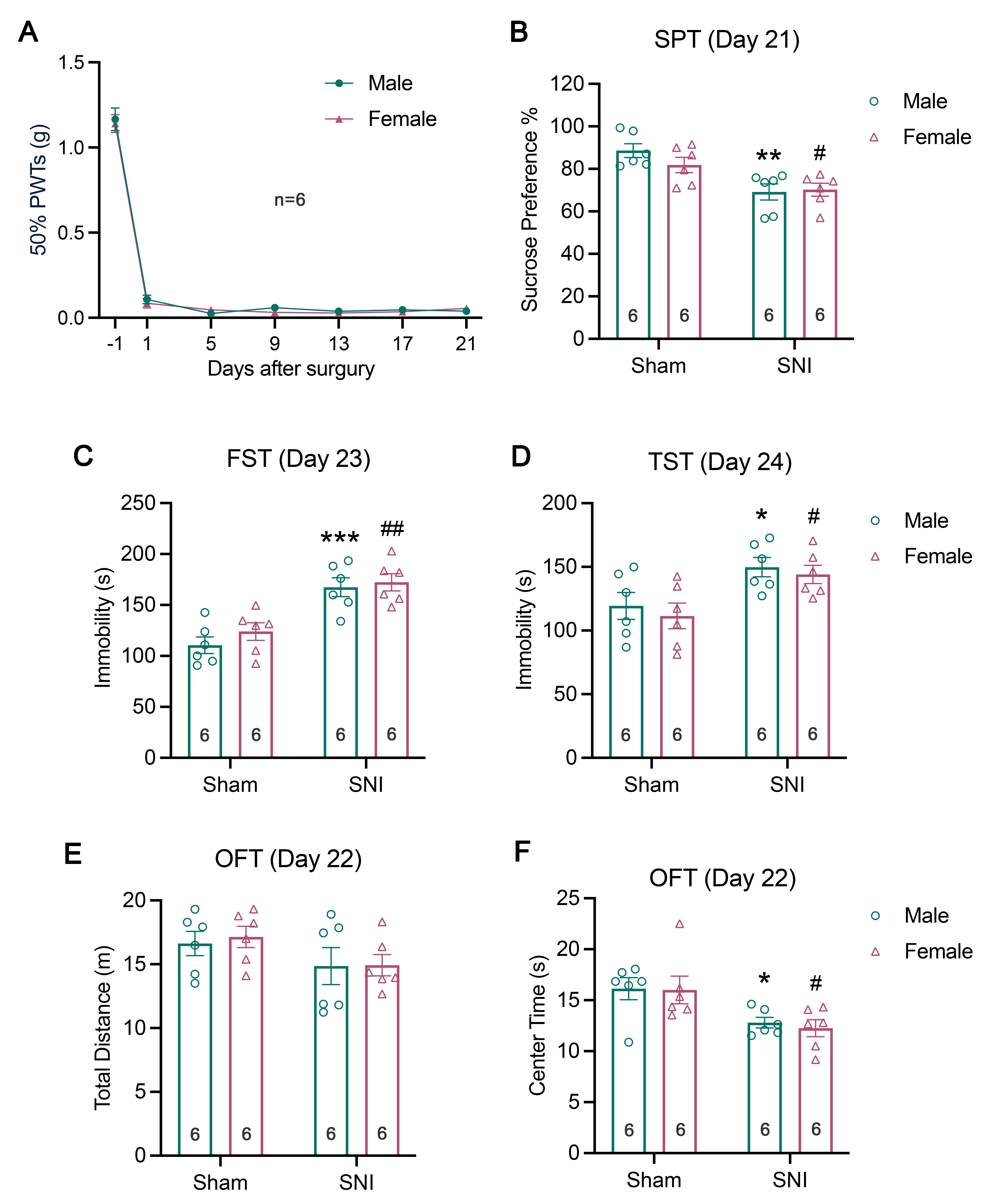

Supplement: Supplementary file 2 — Figure S1 Chronic pain sensation and comorbid depression‐like behaviors of male and female mice. (A) The pain threshold tested on indicated days after the surgery, calculated as 50% PWT. Error bars represent SEM, n = 6. (B–F) Performance of mice with sham or SNI surgery in SPT (B), FST (C), TST (D), and OFT (E, F) on days 21–24 after the surgery. *p < 0.05; **p < 0.01; ***p < 0.001 versus sham male mice. # p < 0.05, ## p < 0.01 vs. sham female mice. Error bars represent SEM, n = 6. Statistical analysis was performed using a two‐way ANOVA. [file CNS-31-e70311-s001.tif]

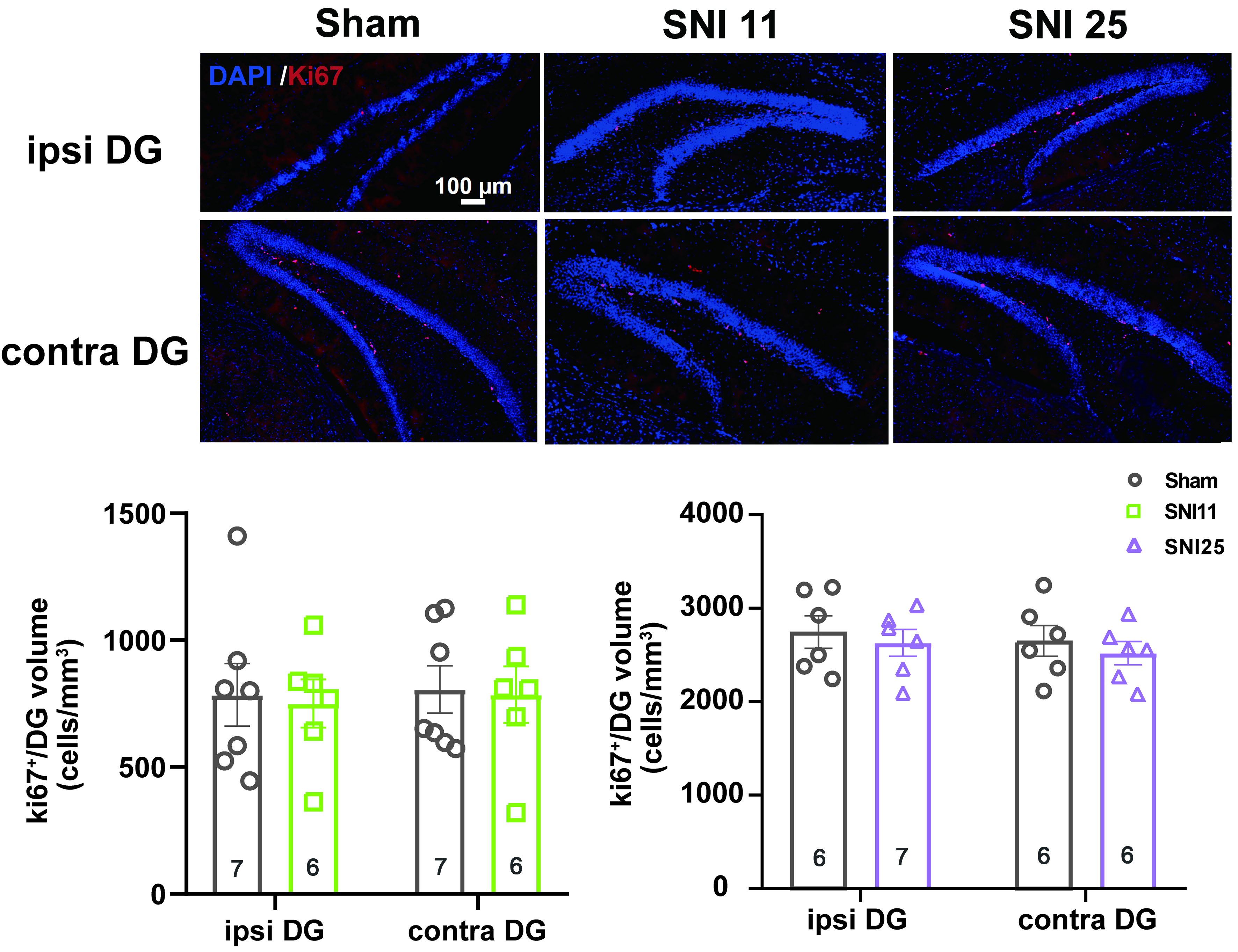

Supplement: Supplementary file 3 — Figure S2 Effects of SNI surgery on the proliferation of hippocampal NSCs. (A) The ipsilateral and contralateral ventral DG of mice with or without SNI surgery were examined by using antibodies against Ki67, the index for cell proliferation, on indicated days. Red: Ki67; Blue: DAPI. Images represent at least six individual animals with similar results. Scale bar: 100 μm. (B) Quantification of Ki67+ cells in the ventral DG. Error bars represent SEM, n ≥ 6. Statistical analysis was performed using a one‐way ANOVA. [file CNS-31-e70311-s004.tif]

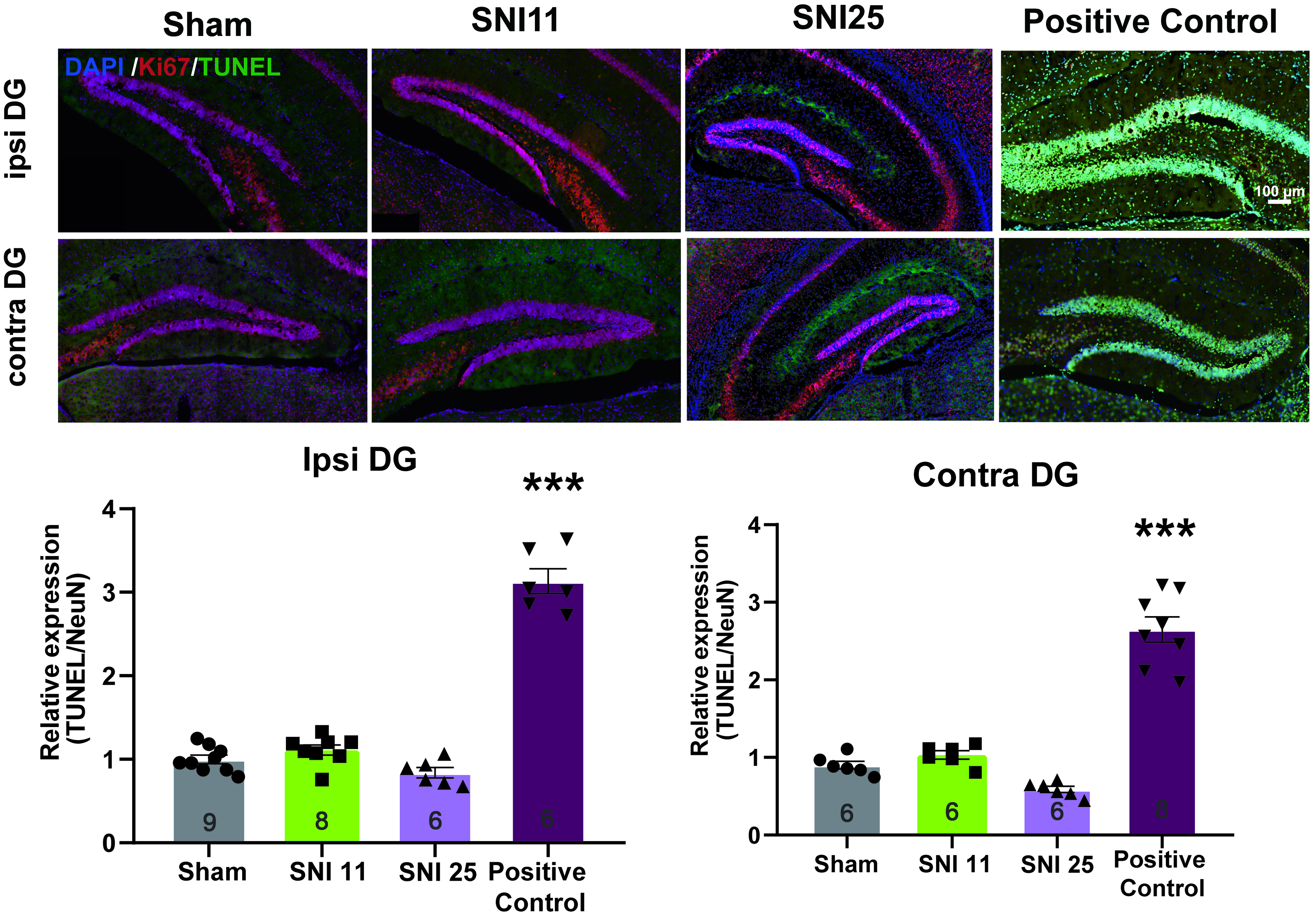

Supplement: Supplementary file 4 — Figure S3 Effects of SNI surgery on the apoptosis of newborn neurons in the ventral DG. (A) Cell apoptosis in the ipsilateral and contralateral ventral DG of mice with or without SNI surgery on indicated days was examined by TUNEL assay. Red: Ki67; Green: TUNEL; Blue: DAPI. Images represent at least six individual animals with similar results. Scale bar: 100 μm. (B) Quantification of cell apoptosis by comparing the average fluorescence of TUNEL and NeuN, calculated as the TUNEL/NeuN ratio. Error bars represent SEM, n ≥ 6. Statistical analysis was performed using a one‐way ANOVA. [file CNS-31-e70311-s002.tif]
