# Supplementary material for: MeCP2 Modulates Depression‐Like Behaviors Comorbid to Chronic Pain by Regulating Adult Hippocampal Neurogenesis
Source: CNS Neurosci Ther. 2025 Apr 7;31(3):e70311. doi: 10.1111/cns.70311 (PMC11974449; doi:10.1111/cns.70311)
Supplement: Supplementary file 5 — Table S1 Antibodies. [file CNS-31-e70311-s005.docx]

**Table S1 Antibodies**

**Primary Antibodies**

**Immunoblotting**

| Antibodies (Abcam) | Cat. No. | Conditions |
| --- | --- | --- |
| Anti-GFAP  Anti-Doublecortin  Anti-MeCP2 [4B4]  Anti-β-actin  Anti-Nestin [SP103] | ab7260  ab18723  ab252840  ab8227  ab105389 | Rabbit polyclonal IgG, 1:1000  Rabbit polyclonal IgG, 1:1000  Mouse monoclonal IgG, 1:1000  Rabbit polyclonal IgG, 1:3000  Rabbit monoclonal IgG, 1:1000 |

**Immunohistochemistry**

| Antibodies (Abcam) | Cat. No. | Conditions |
| --- | --- | --- |
| Anti-NeuN [EPR12763]  Anti-BrdU [BU1/75 (ICR1)]  Anti-Ki67 | ab177487  ab6326  ab15580 | Rabbit monoclonal IgG, 1:100  Rat monoclonal IgG, 1:100  Rabbit polyclonal IgG, 1:1000 |

**Secondary antibodies**

**Immunoblotting**

| Antibody (Bio-rad) | Cat. No. | Condition |
| --- | --- | --- |
| Anti-mouse IgG (H/L): HRP  Anti-rabbit IgG (H/L): HRP | 0300-0108  5196-2504 | Goat polyclonal IgG, 1:3000 |

**Immunohistochemistry**

| Antibodies (ThermoFisher) | Cat. No. | Condition |
| --- | --- | --- |
| Alexa Fluor® plus 647 anti-rabbit IgG  Alexa Fluor® plus 594 anti-rabbit IgG  Alexa Fluor® plus 594 anti-rat IgG  Alexa Fluor® plus 488 anti-mouse IgG  Alexa Fluor® plus 488 anti-rat IgG | A32733  A32740  A48264  A32723  A48262 | Goat polyclonal IgG, 1:3000  Goat polyclonal IgG, 1:3000  Goat polyclonal IgG, 1:1000  Goat polyclonal IgG, 1:3000  Goat polyclonal IgG, 1:1000 |
